# Range expansion, habitat use, and choosiness in a butterfly under climate change: Marginality and tolerance of oviposition site selection

**DOI:** 10.1002/ece3.7202

**Published:** 2021-02-11

**Authors:** Youri Martin, Nicolas Titeux, Hans Van Dyck

**Affiliations:** ^1^ Behavioural Ecology and Conservation Group Earth and Life Institute UCLouvain (Université Catholique de Louvain) Louvain‐la‐Neuve Belgium; ^2^ Observatory for Climate Environment and Biodiversity Environmental Research and Innovation Department Luxembourg Institute of Science and Technology Belvaux Luxembourg

**Keywords:** butterfly, ecological specialization, habitat selection, *Lycaena dispar*, plant–insect interaction, range edge, thermal ecology

## Abstract

Poleward range shifts under climate change involve the colonization of new sites and hence the foundation of new populations at the expanding edge. We studied oviposition site selection in a butterfly under range expansion (*Lycaena dispar*), a key process for the establishment of new populations. We described and compared the microhabitats used by the species for egg laying with those available across the study sites both in edge and in core populations. We carried out an ecological niche factor analysis (ENFA) to estimate (1) the variety of microhabitats used by the butterfly for egg laying (tolerance) and (2) the extent to which these selected microhabitats deviated from those available (marginality). Microhabitat availability was similar in edge and core populations. Ambient temperature recorded at the site level above the vegetation was on average lower at core populations. In contrast with what is often assumed, edge populations did not have narrower microhabitat use compared to core populations. Females in edge populations even showed a higher degree of generalism: They laid eggs under a wider range of microhabitats. We suggest that this pattern could be related to an overrepresentation of fast deciding personalities in edge populations. We also showed that the thermal time window for active female behavior was reduced in edge populations, which could significantly decrease the time budget for oviposition and decrease the threshold of acceptance during microhabitat selection for oviposition in recently established populations.

## Graphical abstract

Contrary to what is often assumed, edge populations did not have narrower habitat use compared to core populations. In the butterfly *Lycaena dispar*, females in edge populations showed higher degree of generalism; they laid eggs under a wider range of conditions.

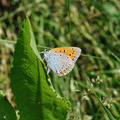

## 1 INTRODUCTION

In a wide variety of organisms, there is evidence of poleward range shifts that are associated with climate warming (Chen et al., 2011; Scheffers et al., 2016). In this context, dispersal may lead to the successful colonization of new sites beyond former range limits. Dispersive individuals that establish new populations at the moving range edge are likely to have specific phenotypes and genotypes (Hassall et al., 2014; Travis et al., 2013). If so, range shifts may result into non‐random spatial redistribution of phenotypes and genotypes at edge populations for adaptive or non‐adaptive reasons (Edelsparre et al., 2014).

Several comparative studies have focused on differences in movement‐related traits between newly founded populations at the edge and well‐established populations at the core (Hill et al., 2011). Examples include higher allocation to morphological traits (e.g., higher muscle mass) and physiological traits (e.g., higher energy metabolism) at edge populations compared to core populations (Hill et al., 1999; Therry et al., 2014).

Dispersal should, however, not be considered independent of other life‐history traits, which forms the rationale of the concept of dispersal syndromes (Buoro & Carlson, 2014; Pruitt et al., 2011; Stevens et al., 2014). In butterflies, for example, dispersal is integrated in life‐history traits that relate to fecundity and ecological specialization and also to variation in functional morphology (Stevens et al., 2012). Dispersive individuals may also represent a biased subsample at the behavioral level compared to the pool of behavioral personalities in well‐established populations (Cote et al., 2010; Quinn et al., 2011). The study of animal personality is one of the fastest‐growing areas of research in behavioral and evolutionary biology (Carere & Maestripieri, 2013); it refers to consistent between‐individual differences in behavior that persist through time or across situations or contexts (Bell, 2007). Assuming specific behavioral profiles for dispersing and colonizing individuals, newly founded populations at the expanding edge may deviate in the average behavioral profile from populations at the range core. Some butterfly species were for instance found to shift host‐plant preference and habitat association during range shifts (Braschler & Hill, 2007; Davies et al., 2006; Thomas et al., 2001).

Butterflies are popular model organism to study range expansion under climate change because these flying heliotherms are highly responsive to temperature and climatic conditions (e.g., Breed et al., 2013; Mills et al., 2017; Parmesan & Yohe, 2003). Most studies assume narrower habitat use at the range edge compared to the core (Oliver et al., 2012; Shreeve et al., 1996; Thomas et al., 1999; Wilson et al., 2010), but this assumption has rarely been tested explicitly. It is difficult to know whether such ecological differences are caused by ecological factors (e.g., habitat availability) or by organism‐related, evolutionary factors (e.g., behavioral differences between populations). Testing for differences in habitat use between newly colonized edge populations and well‐established core populations is helpful to gain insight into the behavioral mechanisms of range expansion (Bennie et al., 2013).

In this paper, we addressed the issue of habitat use in a butterfly under range expansion by focusing on microhabitats used for egg laying and by taking explicitly into account the availability of such microhabitats. We selected a study system with a limited difference in latitude between the current edge and the core of the species range (*c*. 100 km), but with a confirmed difference in population age (colonized since <5 years vs. at least >100 years). We adopted a resource‐based approach to describe the microhabitats required for the oviposition of *Lycaena dispar* (Dennis et al., 2003). This functional, organism‐centered habitat approach is based explicitly on the essential and specific ecological resources required to survive and reproduce, and differs conceptually from a structural habitat approach based on general vegetation types (Dennis et al., 2006; Van Dyck, 2012). Ecological resources include both consumables (e.g., nectar and host plants) and utilities (e.g., microclimate).

We focused on oviposition site selection in three edge and three core populations by comparing the consumables and utilities locally available with those actually used for oviposition. This allowed us testing whether there is support for narrower oviposition‐related microhabitat selection at the edge caused by limited resource availability, or alternatively, by organism‐related differences, independent of environmental conditions in the edge or core populations.

## 2 METHODS

### 2.1 Study system

We used the Large Copper butterfly (*Lycaena dispar*) as study model. In Central and North‐East Europe, its range edge is shifting northward in response to regional climate warming (Strausz et al., 2012). Further expansion is predicted under future climate change projections (Martin et al., 2013). The species is primarily confined to wetland areas (marshes, grasslands, pastures and wastelands) (Lai & Pullin, 2004; Strausz et al., 2012). *Lycaena. dispar* is a bivoltine species and adults fly in late spring (mid‐May to June) and summer (August to mid‐September). Eggs are laid on *Rumex* plant species only. We studied the subspecies *Lycaena dispar rutilus* that reaches its current northern range limit in Western Central Europe (South Belgium and Luxembourg). *Lycaena. dispar* did not occur in this region before 1947 (Rémont, 1952), but its range expanded though rapidly these last 30 years.

We selected three study sites with distinct butterfly populations at the range edge (N 49.68, E 5.55; N 49.70, E 5.98; N 49.69, E 6.36) where *L. dispar* settled less than five years before the study (hereafter “edge populations”). Three other study sites with distinct populations were selected 100 km to the south within the well‐established part of the range in France (N 48.76, E 5.81; N 48.83, E 5.97; N 48.82, E 6.84) where the species’ presence has been documented since >100 years (hereafter ‘core populations’). Study sites were selected to have similar humid grassland vegetation with the same species of host and nectar plants. The three species of the most frequently used host plants (*Rumex crispus, R. obtusifolius* and *R. conglomeratus*) were present in all the study sites. All the sites were extensively managed by late mowing.

### 2.2 Data collection

Field data were collected during spring and summer of 2011, covering both flight periods of *L. dispar*.

#### 2.2.1 Meteorological data

Based on existing meteorological data (OBS‐gridded dataset provided by the ECA&D project; Haylock et al., 2008), we calculated average annual temperatures and average temperatures for the flight period (June 1 to August 31) during the period 1980–2011 and also separately for the year 2011. The meteorological data were extracted at the study site location and averaged across the three sites with edge populations and the three sites with core populations, respectively.

#### 2.2.2 Ambient temperature

Each study site was equipped with a weather station recording ambient temperature every 20 min during the study in June, July and August. Ambient temperature was recorded above the vegetation at typical flight height (at 120 cm) and closer to the ground where oviposition occurred (at 40 cm). The thermal probes of the weather stations (HOBO U23‐002, Onset Computer Corporation) were protected by a solar radiation shield. The weather stations were similarly exposed to open conditions within each study site and placed inside a zone where female butterflies were observed to lay their eggs.

#### 2.2.3 Microhabitats used for oviposition

In each study site, the variety of microhabitats available for butterflies to lay their eggs was described and compared with the range of microhabitats actually used for oviposition. We randomly selected 15 control host plants within each study site, without prior knowledge on plants where eggs were laid, and these control plants were considered to reflect the range of microhabitats available. We recorded nine variables to characterize the ecological resources (consumables and utilities) available around each control plant in a 1 m^2^ square (Table 1). The only measures that were not taken within the square were the distance to the nearest nectar plant and to the nearest other host plant.

**TABLE 1:** Microhabitat variables used to characterize the resources (consumable and utilities) required for the oviposition of the butterfly *L. dispar*

Variables	Description
Temperature at leaf	Temperature recorded by thermal probes at height where the oviposition occurred on the leaf minus the ambient temperature recorded at the same time by the weather station at 120 cm height (°C)
Host plant height	Height of the host plant from the ground to either the flowering stem for plants with inflorescences, or the last leaf in non‐flowering plants (cm)
Host plant consumed	Percentage of the leaves of a host plant consumed by herbivores other than *L. dispar,* estimated visually.
Host plant leaves	Number of leaves of the host plant
Vegetation height	Mean height of the vegetation measured in the four corners of a 1‐m^2^ plot around the focal host plant (cm)
Vegetation cover	Percentage of the vegetation cover in a 1‐m^2^ plot around the focal host plant. It was estimated by assessing visually the percentage of visible bare ground
Host plant density	Number of host plants in a 1‐m^2^ plot around the focal host plant
Host plant isolation	Distance to the nearest host plant, in or outside the 1‐m^2^ plot around the focal host plant (cm)
Distance to nectar	Distance to the nearest nectar source, in or outside the 1‐m^2^ plot around the focal host plant (cm)

Next, we searched exhaustively for host plants with freshly laid eggs (Strausz et al., 2012) in the study site in order to reflect the host plants used for oviposition. Only unhatched and non‐parasitized eggs with a bright white color were considered freshly laid. Eggs were searched in priority within areas where females had been observed flying. The randomly selected control host plants were also checked for presence of freshly laid eggs and were therefore classified as either used or unused host plants depending on the presence of eggs. The same nine variables were recorded for the used host plants as for the control ones.

This procedure was repeated once a week in each study site for three consecutive weeks (visit round: week 1, week 2 and week 3) during each of the two flight periods (generations: spring and summer).

Apart from the ambient temperature recorded at the site level by the weather stations, we also recorded the temperature of the microhabitat. Thermal probes connected to a data logger (HOBO Pendant Loggers UA‐002‐64, Onset Computer Corporation, Bourne, USA) were used to record the temperature at the height of the leaves where eggs were found for used host plants, or at the height where eggs were typically found for control but unused host plants. Temperature of the microhabitat was measured every five minutes during five days. For the first generation, the mean height above ground (mean ± SE) for this measurement was 37.1 ± 0.9 cm (*n* = 186) in edge populations and 40.5 ± 0.9 cm (*n* = 173) in core populations. For the second generation, this height was 27.9 ± 1.1 cm (*n* = 157) and 26.0 ± 0.8 cm (*n* = 176), respectively. We calculated the difference between the temperature of the microhabitat (at leaf level) and the ambient temperature (at the site level) that were recorded simultaneously (Ashton et al., 2009). In this way, we obtained a temperature measure at the microhabitat level that is relevant for oviposition and that captures the thermal gain (or loss) relative to the local ambient temperature.

### 2.3 Data analysis

#### 2.3.1 Ambient temperature recorded in each study site

We calculated the mean ambient temperature recorded in each study site by the weather stations (see above) in two different ways: (a) by making use of all measurements for 24 hr a day, and (b) by taking into account only the time of the day relevant for egg laying when ambient temperature is usually the highest (i.e., in the afternoon between 12 and 17 hr; based on Duffey (1968) and own observations). Ambient temperature recorded at 120 cm and 40 cm were analyzed with linear mixed models relative to the following categorical variables and their two‐way interaction terms: latitude (edge or core), generation (spring or summer) and site (6 study sites) nested within latitude. Models also included visit round (week 1, week 2 or week 3 of the field observations) as a random factor.

Based on Duffey (1968) and on preliminary fieldwork we did in 2010, we used 22°C at 120 cm height as an indicative threshold for ambient temperature under which females do not lay eggs in the field. We calculated a thermally suited time frame for oviposition between 12 and 17 hr as the time available in this period (in minutes) with an ambient temperature >22°C. This was calculated separately for each of the six study sites and for the two generations (from June 1 to June 20 and from August 8 to August 27, respectively).

#### 2.3.2 Microhabitat selection for oviposition

We analyzed the microhabitats used by female butterflies for egg laying in edge and core populations during spring and summer. We used a dataset of 692 1‐m^2^ squares around host plants (*N*_spring_ = 393 and *N*_summer_ = 353) that can be classified as used microhabitats (presence of eggs on the host plant) or available microhabitats (randomly selected host plants that may be used or not, *N* = 692). In order to analyze the same proportion of used versus available microhabitats for each latitude (edge or core) and each generation (spring or summer), we randomly subsampled the data within each study site and each visit round. The prevalence of oviposition (i.e., ratio used vs. available microhabitats) was overall 28%.

First, we compared the range of available microhabitats between edge and core populations. For each generation separately, we performed a principal component analysis (PCA) based on the nine variables characterizing the microhabitats measured in the three edge sites. These data were plotted along the first two principal components. Next, the microhabitats variables measured in the three core sites were over‐plotted in the PCA space built with the edge sites. We calculated the PC‐loadings of the microhabitat variables from the core sites based on the PCA computed with the microhabitat variables measured at the edge sites. Independent of habitat use, these PCA plots allowed exploring for environmental differences in consumables and utilities available for oviposition between the edge and the core sites.

Second, microhabitat selection for oviposition was analyzed separately for the four combinations of latitude and generation by Ecological Niche Factor Analysis (ENFA) (Basille et al., 2008; Hirzel et al., 2002). ENFA is a multivariate analysis that measures habitat selection by assessing the extent to which the realized ecological niche of a species deviates from the habitat conditions that are available on average for the species across a study area (Basille et al., 2008). This analysis identified a first, major axis describing the ecological marginality of the species (i.e., difference between used and available habitat conditions) and a second, uncorrelated axis describing its tolerance (i.e., diversity of conditions used by the species). We used the ENFA framework to analyze oviposition site selection at the microhabitat level. In our study, marginality values could range between 0 (no difference between the microhabitats around all available host plants in the study sites and the microhabitats that were used for oviposition) and 1 (high difference). The tolerance reflected the variety of microhabitats used for oviposition relative to the whole range of microhabitats available around the host plants (Table 1). The tolerance index varied from 0 (high specialization) to 1 (high tolerance).

The ENFA analysis was repeated 10 times with 30% of random bootstrapping between microhabitats available and used for oviposition to estimate confidence intervals (Hirzel et al., 2002). The microhabitat variables were normalized by Box–Cox transformation before running the ENFA algorithm with Biomapper version 4.0.7.373 (Hirzel et al., 2007). The marginality and tolerance indexes were analyzed relative to latitude (edge or core) for both generations separately using linear models (R software version 3.0.2).

## 3 RESULTS

### 3.1 Meteorological conditions at the edge and the core

Ambient temperature during the flight period of *L. dispar* was up to 1.35°C cooler at the edge than at the core study sites (Table 2).

**TABLE 2:** Mean meteorological temperatures at edge and core sites based on meteorological records from the OBS‐gridded dataset provided by the ECA&D project (Haylock et al., 2008) for the period 1980–2011 and for the year 2011. Values were averaged across study sites in edge and core (± *SD*), respectively. Averages were both calculated on an annual basis, but also within the flight period of *L. dispar* (June 1 to August 31)

	Annual mean		Flight period	
Latitude	1980–2011	2011	1980–2011	2011
Edge	9.47 ± 0.24	10.67 ± 0.03	17.13 ± 0.36	16.77 ± 0.16
Core	10.36 ± 0.02	11.6 ± 0.08	18.31 ± 0.11	18.12 ± 0.07
∆ Core – Edge	0.86	0.93	1.18	1.35

### 3.2 Ambient temperature at site level

**FIGURE 1:**
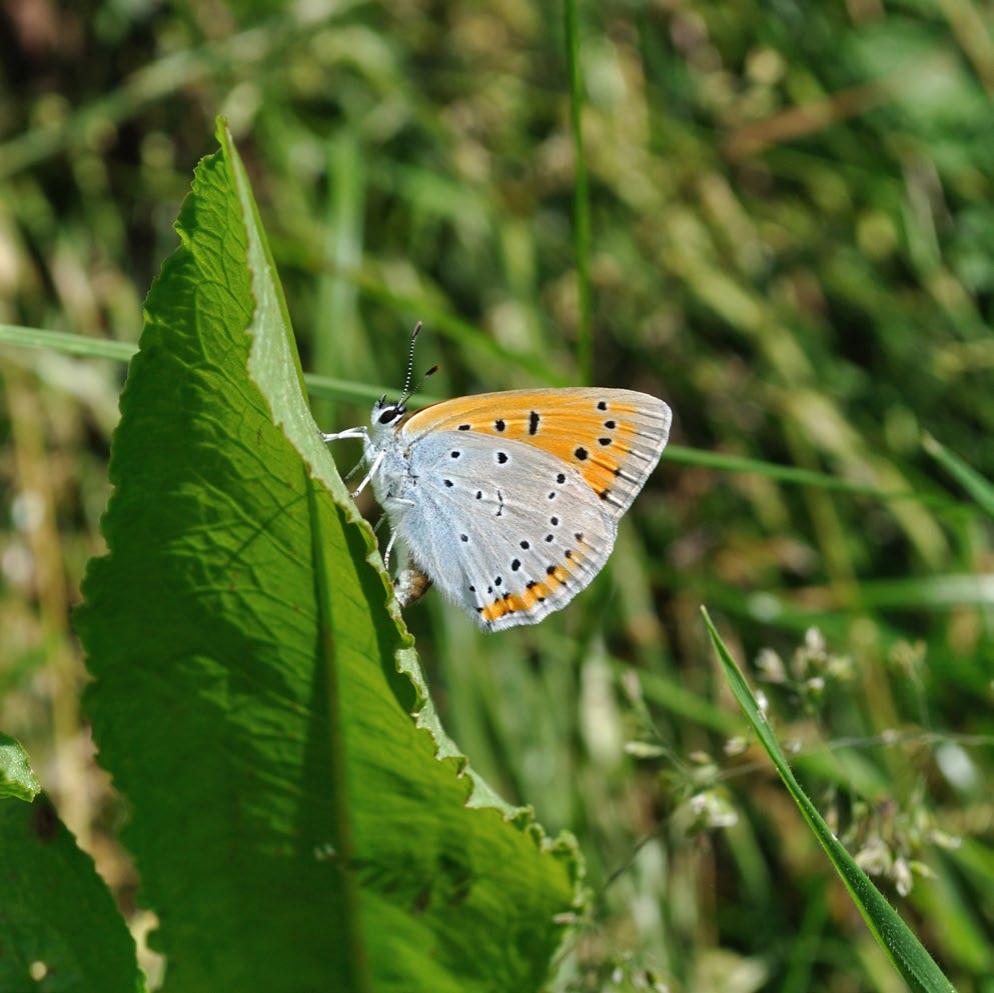
Microhabitats used for egg laying were studied in recently colonized edge populations and well‐established core populations of the large copper butterfly (*Lycaena dispar*). This is a female laying an egg on *Rumex crispus*. Photo taken by Youri Martin

Ambient temperature as recorded in the study sites at flight height (120 cm) was significantly cooler at the edge than at the core sites (Table 3). The difference was stronger for ambient temperature in the afternoon when butterflies lay their eggs. Table 4 summarizes the measured temperatures and differences in temperature relative to latitude and generation. Overall, ambient temperature was 1.36°C lower in the edge sites compared to the core sites (spring and summer data pooled). The temperature was significantly higher at 40 cm than at 120 cm, particularly in the afternoon (Tables 3 and 4). There was also an obvious generation effect as it was warmer in summer than in spring (Table 3). There was no effect of study site nested within latitude, which indicates that ambient temperatures of the three study sites at the same latitude (either edge or core) were similar, independent of generation and of the height of the measurement (Figure 1).

**TABLE 3:** Results of multiple linear mixed regression models analyzing ambient temperature (recorded by the weather stations in each study sites) relative to latitude, generation, study site, height of measurement (40 and 120 cm), visit round, and the two‐way interaction effects. Site was nested within latitude, and visit round is a random factor. 40 cm was at the leaves of the host plants; 120 cm represents the typical height of butterfly flight. Analyses were done with temperature recordings (a) all day (24 hr), or (b) in the afternoon only (12 hr–17 hr)

Fi × ed factors	*df*	All day	Afternoon
Latitude	1	11.4***	15.3***
Generation	1	19.6***	33.7***
Site[Latitude]	4	6.4	6.3
Height	1	5.83***	20.4***
Latitude × generation	1	1.9	0
Latitude × height	1	0.5	0.8
Latitude × visit	4	28.9***	33.7***
Generation × visit	2	73.1***	77.0***
Height × visit	2	0	0
Height × generation	1	0.5	0.5
Residuals	51	–	–

Notes

Visit is a random effect explaining, respectively, 2.00 and 2.27 of the variance for All day and Afternoon and with 1.45 and 1.18 for the residual variance.

*** *p* < 0.001, ^**^ *p* < 0.01, ^*^ *p* < 0.05.

**TABLE 4:** Ambient temperatures (°C) recorded by the weather stations in each study site at the height of the vegetation for oviposition (40 cm) and at the height of butterfly flight (120 cm) with indication of the difference between both measures. Values were averaged among sites by latitude (± St. Dev) and calculated with temperature recordings (a) all day (24 hr), or (b) in the afternoon only (12 hr–17 hr)

		Spring		Summer	
		All day	Afternoon	All day	Afternoon
40 cm	Edge	15.2 ± 0.4	22.5 ± 0.4	15.8 ± 0.3	23.9 ± 0.8
	Core	15.5 ± 0.3	23.3 ± 0.4	17.8 ± 0.1	24.7 ± 0.3
	∆ Core – edge	0.34	0.8	1.94	0.8
120 cm	Edge	15.4 ± 0.2	20.8 ± 0.3	16.1 ± 0.4	22.4 ± 0.3
	Core	15.6 ± 0.3	22.0 ± 0.6	17.9 ± 0.2	23.9 ± 0.6
	∆ Core – edge	0.27	1.24	1.81	1.49
∆ 40 – 120 cm	Edge	−0.15	1.73	−0.25	1.47
	Core	−0.08	1.29	−0.12	0.78

The daily time budget for female flight and oviposition varied considerably at 120 cm height (35.8% to 70.0%) and at 40 cm (49.6% to 75.6%) (Appendix Table S1). Female butterflies had reduced time budgets for flight and oviposition in the edge sites compared to the core sites (at 120 cm: *F*_1,4_ = 7.88, *p* < 0.05; spring: decrease of 6.8%; summer: 15.5% and a near‐significant tendency at 40 cm: *F*_1,4_ = 4.70, *p* < 0.1; spring: decrease of 2.8%; summer: 10.8%).

### 3.3 Microhabitat selection for oviposition

**FIGURE 2:**
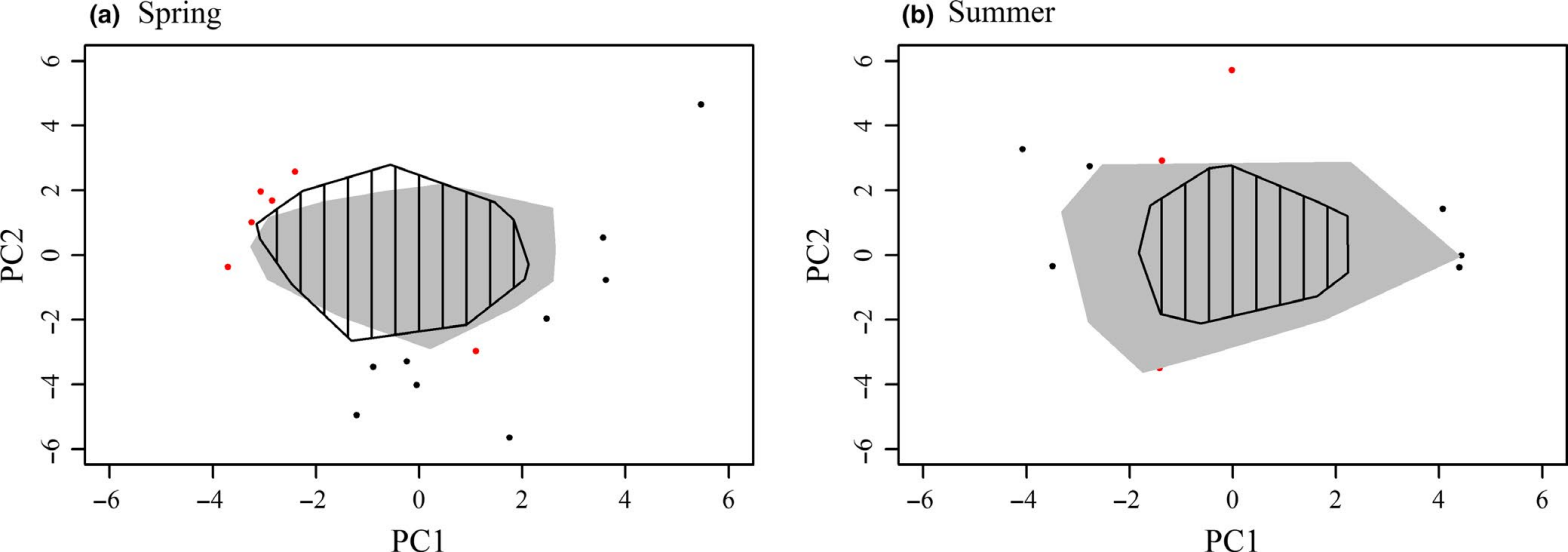
Synthesis of available microhabitats as expressed by the first two axes of the principal component analyses (PCA) based on 9 microhabitat variables (see Appendix Table S2 for descriptive statistics of the PCA) for the spring and the summer generation separately. The environmental space reflects the microhabitats available for the butterfly at the edge (gray) and at the core (dashed) sites. Data on microhabitat available for the butterfly in the edge populations were used to construct the principal component axes. The data on microhabitat available for the butterfly in the range core were then overlaid in the same environmental space based on their PC scores. The polygons were delineated using the “Convex Hull” method and encompassed 95% of the microhabitat availability for the edge (gray) and the core (dashed) populations. Black and red points indicate the remaining 5% of outliners for edge and core populations, respectively

Appendix Table S2 shows the details of the axe loadings resulting from the principal component analysis based on the microhabitat variables. Figure 2 shows the degree of similarity between the microhabitats available for oviposition in the edge and core sites based on the first and second PCA axes. There was no indication of any difference in available microhabitats for oviposition between the edge and core sites along these PCA axes. Hence, except for ambient temperature, all other aspects of microhabitats available for laying eggs were similar in both the edge and the core sites.

**FIGURE 3:**
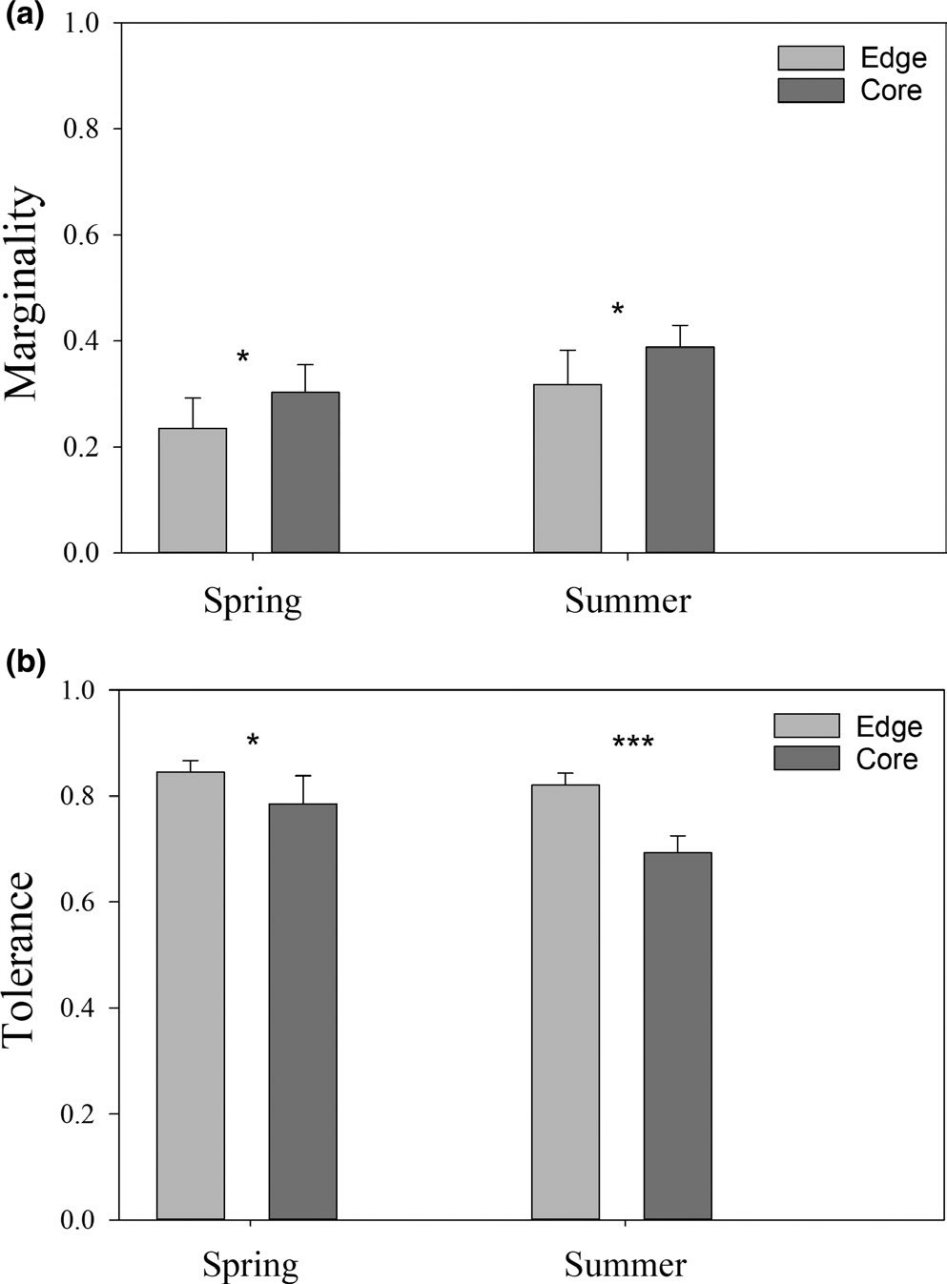
Global marginality (a) and tolerance (b) for used microhabitats relative to available microhabitats in edge populations (light gray) and core populations (dark gray) for the spring and summer generation (± *SD*; based on 10 replicates of the ENFA analysis with 30% random bootstrapping). Levels of statistical significance difference between edge and core. **: *p* < 0.001, **: *p* < 0.01, *: *p* < 0.05

For both generations, ENFA‐based marginality scores were significantly smaller for edge populations compared to core populations (spring: *F*_1,18_ = 7.89, *p* < 0.05; summer: *F*_1,18_ = 8.10, *p* < 0.05) (Figure 3). This indicates that the microhabitats used for oviposition at the edge deviated less from available conditions than they did at the core. In both generations, ENFA‐based tolerance scores were significantly higher at the edge compared to the core (Spring: *F*_1,18_ = 5.98, *p* = 0.024; Summer: *F*_1,18_ = 22.66, *p* < 0.001) (Figure 3). Therefore, butterflies at the core populations made use of a smaller subset of available microhabitats than they did at the edge sites. Details on the contribution of the microhabitat variables to the marginality score are provided in Appendix Figure S1. Appendix Figure S2 shows the marginality of microhabitats use along each of the individual variables. For example, the temperature at leaf was an important variable to explain microhabitat selection for oviposition in spring, especially for edge populations, whereas it was less important in summer (Appendix Figure S1). The difference between the available and used thermal conditions for oviposition at the host plant leaf level shows that eggs were laid in warmer microhabitats than what was available, but the effect was not more pronounced at the edge compared to the core (spring: 0.53°C and 0.50°C, respectively; summer: 0.39°C and 0.81°C, Appendix Figure S2).

## 4 DISCUSSION

We demonstrated significant differences in the selection of microhabitats for oviposition between newly colonized edge populations and well‐established core populations of the large copper butterfly *Lycaena dispar* under range expansion. Ambient temperature was overall lower at the northern edge than at the core of the range, but what really matters to a butterfly are the conditions at the microhabitat level. Contrary to what has often been assumed (e.g., Hill et al., 1999; Oliver et al., 2009; Shreeve et al., 1996; Thomas et al., 1999; Wilson et al., 2010), individuals of the newly colonized, edge populations did not have narrower microhabitat use for egg laying compared to core populations. We even found evidence of the opposite pattern as edge populations have wider, more opportunistic microhabitat use than core populations. This pattern was found in both the spring and summer generations of the butterfly.

Studies on different habitat use between edge and core populations are usually associated with differences in available habitat, but this is rarely quantified explicitly (Thomas et al., 1999). There is also growing evidence of behavioral divergence between core and edge populations (e.g., Gruber et al., 2017; Reim et al., 2018). For example, butterflies were shown to use particular microhabitats or switch host plant preference at the edge because the plants are located in more favorable climatic conditions or are more widespread than in the core of the species range (Braschler & Hill, 2007; Davies et al., 2006; Thomas et al., 2001; Pateman et al., 2012). Consequently, it may be difficult to interpret whether differences in habitat use in edge populations result from environmental factors (e.g., varying availability of consumables and/or utilities), from organism‐related factors (e.g., different behavioral profiles), or from both. Our results showed differences in habitat use for egg laying between recently established edge populations and well‐established core populations where consumables were similarly available. Studies on components of habitat use do not always identify consumables and utilities separately (Dennis et al., 2003, 2006). Organism‐related factors could also play a significant role to explain such a pattern.

Recent work on dispersal and animal personality provides a useful framework for organism‐related factors, also in a context of range shifts (e.g., Gruber et al., 2017). If colonizers were not a random sample of the core populations but individuals of particular behavioral type associated with dispersal, then such behavioral types will become frequent in newly founded edge populations due to the non‐random process of dispersal during range expansion (Cote et al., 2010; Edelsparre et al., 2014; Reim et al., 2018). Such a mechanism could contribute to explaining the observed differences in our study. Higher tendencies (or distances) of dispersal have been associated with aggressiveness and exploratory behavior, which was in turn associated with bold and fast decision making personalities (Chapple et al., 2012; Cote et al., 2010; Quinn et al., 2011). The study of animal personality in the wild contributes in a growing way to our understanding of habitat selection (Clobert et al., 2009; Leclerc et al., 2016). Stevens et al. (2012) indicated that the relationship between dispersal and the evolution of ecological specialization is not always straightforward. Our results on marginality and tolerance scores suggest that the range expansion in *L. dispar* is associated with increased generalism during microhabitat selection for oviposition, a conclusion that is consistent with the results of Lindman et al. (2015).

Although the availability of consumables (e.g., host and nectar plants) was similar between edge and core sites, we found differences for utilities that relate to temperature. Ambient temperature at flight height was on average considerably lower at range edge. Consequently, the time budget for active female behavior (including oviposition) was reduced in edge populations compared to core populations (up to 15%). Berger et al. (2012) calculated that smaller time budget for oviposition could significantly decrease female fitness. Time stress at the edge may alter the choosiness of egg‐laying females as choosiness (i.e., time required to select a high quality host plant) will be traded‐off for against fecundity (i.e., number of eggs laid) at the edge, but not, or less so, at the core of the range (Berger et al., 2012; Doak et al., 2006; Kingsolver, 1983). This is of particular relevance for relatively short‐living, thermophilous species with high egg maturation rate (Berger et al., 2012; Doak et al., 2006) such as *Lycaena dispar* (Lindman et al., 2015). Increased time stress at the range edge may select for individuals which have lower levels of choosiness compared to individuals from the well‐established core populations as a compensatory behavioral mechanism for shorter time budget (Therry et al., 2014). Time constraints can be differentially affected by latitude‐related variation in lifespan, but we have no accurate information on this life‐history trait for our study system.

Since we showed wider niche use for oviposition at the edge compared to the core of the range, which was independent of the ecological consumables (i.e., host plants), we suggest two non‐mutually exclusive mechanisms. First, females at the range edge have a reduced thermal time budgets for flight and oviposition that may facilitate the acceptance of host plants for egg laying. Second, newly colonized populations have overrepresentation of fast deciding personalities that may also facilitate the acceptance of oviposition sites. At this stage, we have no evidence in favor of one of the mechanisms, or in favor of a synergistic effect. Selection on such behavioral phenotypes may promote faster range expansion (Phillips et al., 2010; Reim et al., 2018). Since methods on testing butterfly personality have recently been developed and applied also in the field (e.g., Kaiser et al., 2020, and references therein), further research including personality tests and detailed work on habitat selection of *L. dispar* females during oviposition in both types of populations is now warranted.

Based on our study, we reinforce the need to take into account fine‐scale data on habitat use and ecological resources for a better understanding of the mechanisms behind range shifts under climate change (Chave, 2013; Pincebourde & Woods, 2020; Potter et al., 2013). Fine‐scale data are collected at spatial and temporal scales congruent with the functional environmental relationships of the study organism, including its thermal environment (Bennie et al., 2013; Suggitt et al., 2012; Turlure et al., 2010). Such an approach will help guide in situ management under global change (Greenwood et al., 2016; Turlure et al., 2019).

## CONFLICT OF INTEREST

None declared.

## AUTHOR CONTRIBUTION

**Youri Martin:** Conceptualization (equal); Data curation (lead); Formal analysis (lead); Funding acquisition (equal); Investigation (lead); Writing‐original draft (equal); Writing‐review & editing (equal). **Nicolas Titeux:** Conceptualization (equal); Data curation (supporting); Formal analysis (supporting); Investigation (supporting); Writing‐original draft (equal); Writing‐review & editing (equal). **Hans van Dyck:** Conceptualization (equal); Data curation (supporting); Formal analysis (supporting); Funding acquisition (equal); Writing‐original draft (equal); Writing‐review & editing (equal).

## Supporting information

Appendix S1

## Data Availability

Data are available from the Dryad Digital Repository: https://doi.org/10.5061/dryad.cz8w9gj2q.
