# Supplementary material for: Range expansion, habitat use, and choosiness in a butterfly under climate change: Marginality and tolerance of oviposition site selection
Source: Ecol Evol. 2021 Feb 11;11(5):2336–45. doi: 10.1002/ece3.7202 (PMC7920772; doi:10.1002/ece3.7202)
Supplement: Supplementary file 1 — Appendix S1 [file ECE3-11-2336-s001.docx]

**Appendix**

**Table S1** Thermal time window for female flight activity (including oviposition) of *L. dispar* (± St. Dev.; based on 3 study sites at the edge and the core of the range). Values are the proportion of time in the afternoon (between 12 h and 17 h) with ambient temperature > 22°C either at 40 cm or 120 cm height, expressed as percentages (± St. Dev.)

|  | 40 cm | | 120 cm | |
| --- | --- | --- | --- | --- |
| Latitude | Spring | Summer | Spring | Summer |
| Edge | 49.6 ± 3.8 | 64.8 ± 1.4 | 35.8 ± 0.4 | 54.5 ± 9.8 |
| Core | 52.4 ± 1.2 | 75.6 ± 1.8 | 42.6 ± 3.4 | 70 ± 4.8 |
| ∆ Core - edge | -2.8 | -10.8 | -6.8 | -15.5 |

**Table S2.** Principal component analysis (PCA) based on 9 microhabitat variables for the spring and the summer generation. Table shows the loadings of the variables for the first two PC-axes. The percentage of explained variance by each axis is indicated between brackets.

|  | | Generation | | | | |
| --- | --- | --- | --- | --- | --- | --- |
|  | | Spring | | | Summer | |
|  | PC 1 (22.6 %) | | PC 2 (15.5 %) | PC 1 (34.7 %) | | PC 2 (14.2 %) |
| Vegetation height | -0.538 | | 0.12 | -0.434 | | 0.084 |
| Vegetation cover | -0.412 | | 0.059 | -0.410 | | 0.014 |
| Host plant isolation | -0.083 | | -0.675 | -0.211 | | -0.627 |
| Host plant density | 0.282 | | 0.496 | 0.333 | | 0.331 |
| Host plant leaves | -0.054 | | -0.278 | 0.085 | | 0.284 |
| Distance to nectar | -0.157 | | -0.321 | 0.427 | | 0.096 |
| Host plant consumed | 0.156 | | 0.037 | -0.176 | | 0.519 |
| Host plant height | -0.518 | | 0.154 | -0.393 | | 0.335 |
| Temperature at leaf | 0.362 | | -0.267 | 0.332 | | -0.128 |

**Figure S1**

**
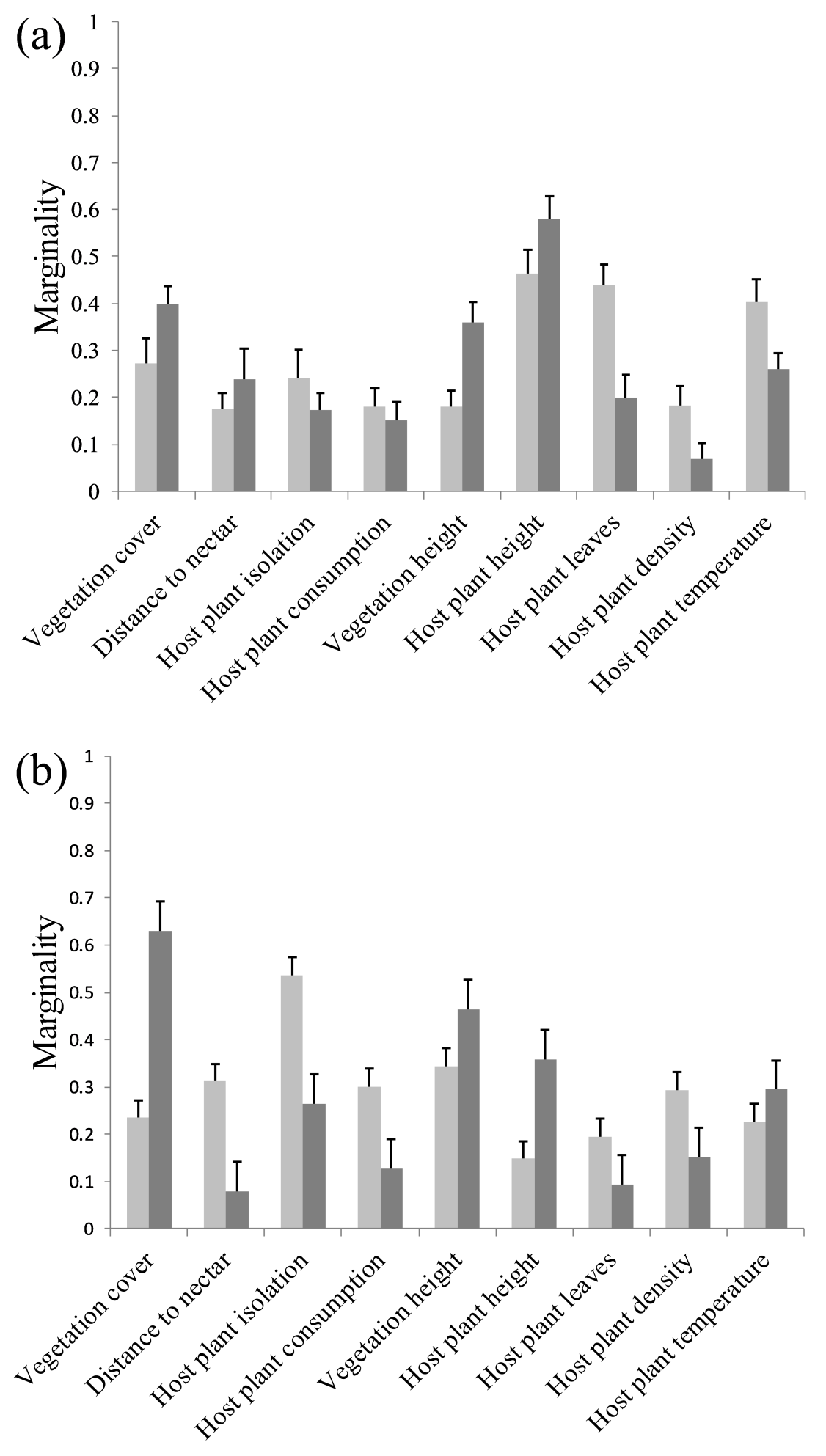
**

**Figure S1.** Contribution of the 9 microhabitat variables to the marginality axis of the ENFA-analysis for (a) the spring generation and (b) the summer generation in edge (light grey) and core (dark grey) populations (± St. Dev.; based on the 10 replicates with random bootstrapping).

**Figure S2**

**
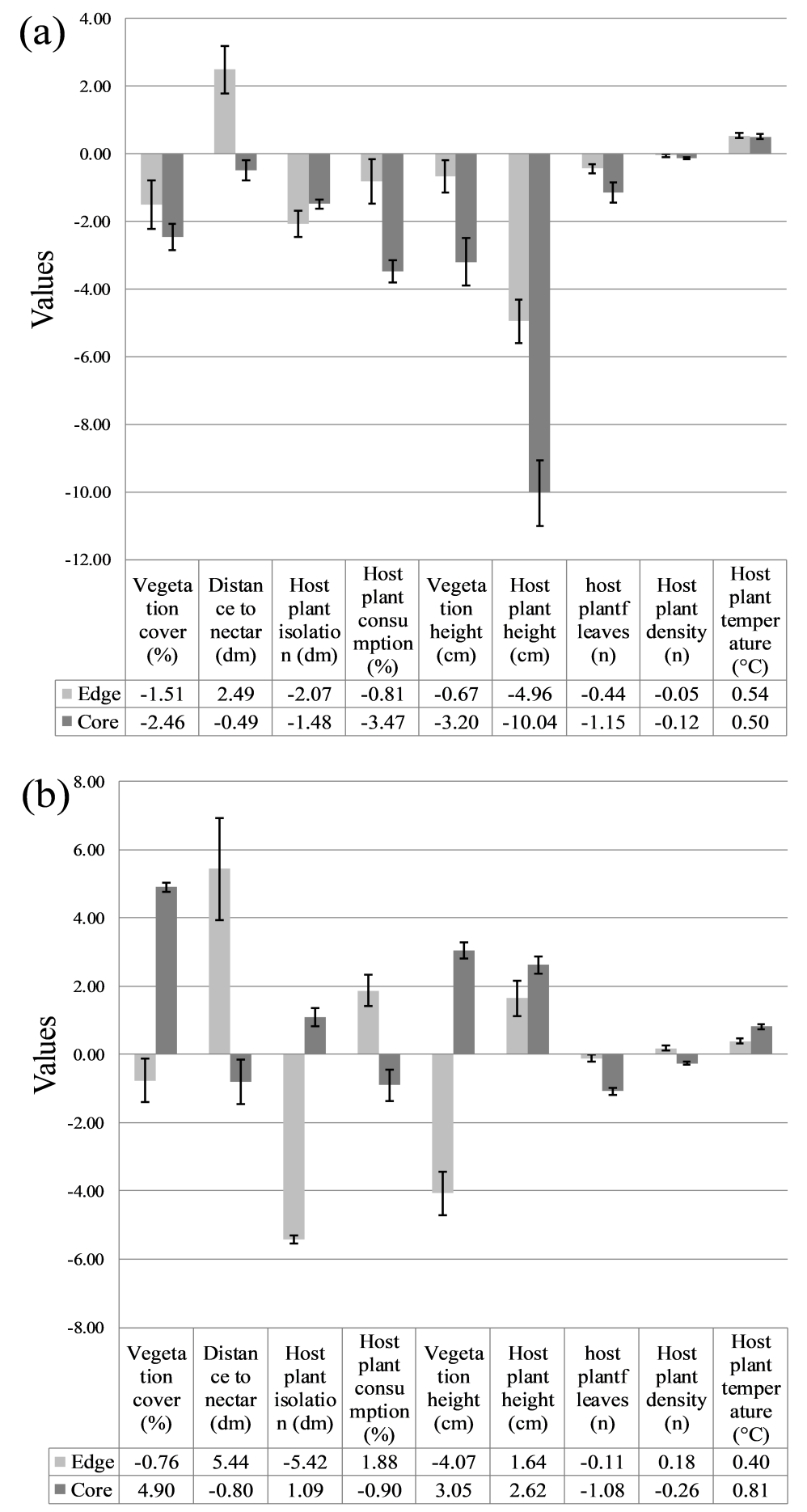
**

**Figure S2** Microhabitat conditions selected for oviposition by *L. dispar* relative to the available conditions for (a) the spring generation and (b) the summer generation at the edge (light grey) and at the core sites (dark grey) (± St. Dev.; based on the 10 replicates of random bootstrapping). The value for each microhabitat variable corresponds to the difference between selected and available conditions. Positive values indicate preference by the butterfly. Values close to zero indicate no particular preference within the available conditions.
